# Nutritional Knowledge and Attitudes among Physician Interns Graduated from King Abdul-Aziz University, Jeddah, Saudi Arabia

**DOI:** 10.3390/healthcare10091788

**Published:** 2022-09-16

**Authors:** Zainab Bawazir, Amani Alrasheedi, Buthaina Aljehany

**Affiliations:** Food and Nutrition Department, Faculty of Human Sciences and Design, King Abdul Aziz University, Jeddah 23213, Saudi Arabia; aalrasheedi@kau.edu.sa (A.A.); baljehany@kau.edu.sa (B.A.)

**Keywords:** physicians, knowledge, attitude, nutrition curriculum

## Abstract

This study aimed to assess the nutritional knowledge and attitudes of physician interns graduated from King Abdul-Aziz University. A cross-sectional study was conducted among 100 physician interns who graduated in 2019, 2020, and 2021. An online self-administered questionnaire was used to collect data. A modified version of a validated questionnaire was used and provided to the participants in both Arabic and English versions, of which participants could choose one. This questionnaire consisted of three sections, including demographic data (five questions), attitude (47 questions), and knowledge (40 questions). A total of 100 (54 female and 46 male) participants completed the questionnaire. The nutrition and diabetes axis had the highest percentage of correct answers among the knowledge axes (55.6%), while the percentage of correct answers for the axis of nutrition and heart disease was the lowest (44%). There were no significant differences among most of the knowledge axes according to sex. However, there were significant differences between the averages of the answers of the male and female groups in the axis of nutrition and obesity only, where the male group had more correct answers than the female group. The average attitude of the study participants was almost good, with slight differences between males and females. A total of 51% of the participants were satisfied with the medical nutrition curriculum. The inadequacy of the nutritional curriculum among medical students was reflected in their satisfaction with their college programs and thus in their knowledge, attitudes, and confidence in providing nutritional counseling to patients. Intern physicians need to improve their clinical nutrition knowledge and skills to be able to provide patients with appropriate nutrition advice.

## 1. Introduction

Nutritional care by health professionals has been shown to increase nutritional intake and quality of life for hospitalized patients who suffer from malnutrition. Doctors and dietitians are the main providers of nutritional care within a hospital. The nutritional knowledge of these professions and the effectiveness of nutritional education interventions have been investigated in Western countries [1]. Primary health-care staff must have sufficient knowledge to determine whether patients are at nutritional risk so that they can offer appropriate intervention [2].

Malnutrition in hospitalized patients contributes to morbidity, mortality, and long-term hospitalization and reduces the quality of life. Nutritional care is often neglected in clinical practice, despite the dramatic increase in the prevalence of nutrition-related diseases, as well as the increased awareness of the risks of both overnutrition and malnutrition over the past two decades [3]. Cardiovascular diseases are the main cause of death at the global level, and the mortality rate of these diseases increased to 42% between 1990 and 2003 worldwide. Poor diet quality is one of the most important factors leading to these diseases. Urbanization and economic growth have also contributed to an increase in the consumption of processed and unhealthy foods and the reduction of healthy foods in diets [4]. Previous studies conducted on patients in public hospitals have shown that poor nutritional status is associated with worse clinical outcomes and that appropriate nutritional interventions can improve these outcomes. Indeed, nutrition plays a major role in the prevention and treatment of many of the main causes of chronic disease and premature death [2].

However, health professionals face barriers in providing nutritional care to patients. These barriers include poor nutritional knowledge, low confidence, and negative attitudes toward integrating nutrition with patient care [5]. The lack of time spent with patients, lack of financial return, minimal education, and lack of comfort in providing nutritional advice are also factors that are taken into consideration [4]. The primary care providers are expected to be major providers of nutritional information, especially given the data indicating that patients consider physicians to be one of the most reliable sources of nutritional information [6].

A limited number of studies have assessed the nutritional knowledge of physicians. Among the few studies on physicians’ nutritional knowledge, low scores were reported in Canada, The US, Taiwan, Saudi Arabia, and Turkey [7]. Results of a survey on medical students in northwestern Iran found that more than half the participants had poor nutritional knowledge and female students have more nutritional knowledge than males. Still, in general, they all have low nutritional knowledge [7,8]. Several studies in the US and other countries have evaluated the status of nutritional education in the medical curriculum and found that the level of nutritional education was insufficient [8,9]. In another study, most of the participants reported that they received minimal or no nutritional education during their medical studies. A Lebanese study reported that doctors showed a positive attitude toward nutritional counseling; however, it described their nutritional education as poor [10].

An assessment of knowledge and attitudes regarding nutrition among future physicians may increase our understanding of the level of nutritional education in the Kingdom of Saudi Arabia (King Abdul-Aziz University). It could also provide baseline data for educators, nutrition practitioners, health professionals, education researchers, and policymakers to inform efforts toward medical curriculum improvements. Therefore, this study aimed to assess the nutritional knowledge of trained physicians at King Abdul-Aziz University. It also aimed to determine the level of discrepancy between nutrition knowledge and attitudes toward nutrition.

## 2. Material and Methods

A cross-sectional study was conducted on physician interns (male and female) who graduated from the Medical School at King Abdul-Aziz University in 2019, 2020, and 2021. An online self-administered questionnaire was used to collect data over a 13-month period: from August 2020 to September 2021. Ethics approval was obtained from the Research Ethics Committee of the Unit of Biomedical Ethics and the Research Ethics Committee of KAU Faculty of Medicine (228-16). The recruitment of potential participants was accomplished after explaining the objectives of the study by the interviewers. Participation was voluntary and verbal consent was acquired from each participant. Confidentiality was maintained for all of the participants. Questionnaires were sent to the target group. Informed consent was obtained from every participant individually. The data were obtained from the subjects by online self-administrated questionnaires that took approximately 10–15 min. A modified version of a validated questionnaire was used in this study [5]. The original questionnaire was translated and culturally adapted to Arabic, and showed adequate reliability and validity. The questionnaires were provided to the participants in both Arabic and English versions, and the participants were given the chance to choose one of the versions. This questionnaire consisted of three sections: demographic data (five questions), attitudes (47 questions), and knowledge (40 questions). The first part (nutrition knowledge) was developed, and questions selected from a standardized nutrition textbook were used on the basis of face validity [11]. Nutrition knowledge was assessed by calculating the percentage of correct responses to 40 multiple-choice questions. Questions were divided into four sections: nutrition and diabetes (nine questions); nutrition and obesity (13 questions); nutrition and heart disease (seven questions); and nutrition and other diseases (11 questions). The second part (Nutritional attitudes) was assessed using the previously validated Nutrition in Patient Care Survey (NIPS) (McGaghie et al., 2001). Possible responses ranged from “strongly disagree” to “strongly agree” on a five-point Likert scale. The third part (Demographic data) consisted of few basic questions, such as age, height, weight, sex, marital status, and year of graduation. Finally, the participants were asked to rate the modules they had completed in the Nutrition curriculum in medical school.

To determine attitudes, arithmetic values were assigned to the categories (strongly agree = 5; agree = 4; neutral = 3; disagree = 2; strongly disagree = 1), and then the answers were classified into five equal range levels. To make the statistical analysis easier, the attitude statements were divided into positive and negative attitude statements.

Data analysis was carried out using the Statistical Package for Social Sciences (SPSS ver. 22.0, IBM, Armonk, NY, USA). Descriptive statistics were applied (i.e., frequency and percentage for categorical, and mean and standard deviation for continuous variables), and the chi-square test and correlation coefficients were used for the assessment of the association between the studied variables. A *p*-value lower than 0.05 was considered statistically significant.

## 3. Results

**Figure 1:**
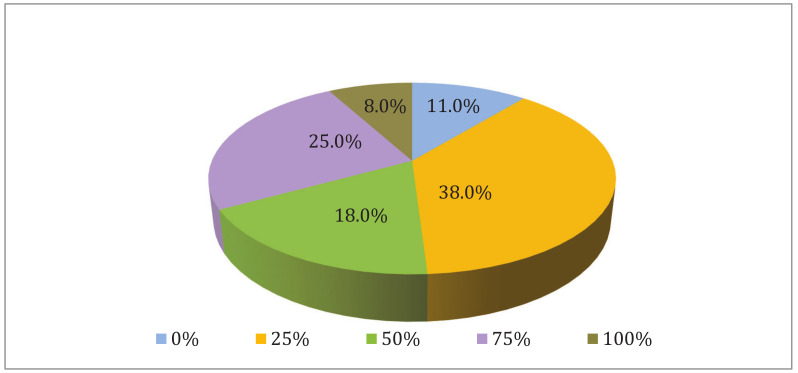
Distribution of the study participants according to evaluation of the Nutrition curriculum in Medicine.

The total sample size in this study was 100 participants (54 female and 46 male), randomly selected. The distribution of the study participants according to demographic variables and body mass index (BMI) is shown in Table 1 and Table 2. Figure 1 shows the distribution of the study participants according to their evaluation of the Nutrition curriculum in Medicine. Approximately half of the study participants (51%) were satisfied with the nutrition curriculum to a level above 50% as follows: 25.0% of the individuals with 75% satisfaction, 18.0% of the individuals with 50% satisfaction, and 8.0% of the individuals with 100% satisfaction. However, the remaining 49% of the participants were not satisfied with the curriculum, considering that 38.0% of the individuals rated the curriculum with 25%, and 11.0% rated it 0%.

Knowledge assessment was divided into four main sections: nutrition and diabetes; nutrition and obesity; nutrition and heart diseases; and nutrition and other diseases. The study participants’ responses to the questions on the nutrition and diabetes axis showed that correct answers accounted for 55.6% of the questions, and wrong answers accounted for 44.4%, which illustrated the difference in their knowledge about this subject. The question that most of the participants answered correctly (81.0%) was about the complication of prednisone therapy that requires nutritional intervention. Most of the students (76.0%) did not know the correct answer to the question on how many grams of carbohydrate are contained in one serving unit.

The various answers of the study participants regarding nutrition and obesity showed that almost half the study participants (53.9%) gave the correct answers, while 46.1% had wrong answers. The most often correctly answered question was the one about the medical nutritional therapy of obstructive sleep apnea (OSA) patients (75.0%), while the reason for losing weight in Dr. Atkins’ diet had the highest proportion of wrong answers (78.0%).

The answers of the study participants to the questions related to the axis of nutrition and heart diseases showed that there were 44.1% of the students with correct answers and 55.9% with wrong answers. The most frequently correctly answered question (59%) was the one about the medical nutrition therapies to decrease hypertension, while LDL cholesterol level that requires dietary intervention was the most often wrongly answered question (89.0%).

The study participants answered the questions on the nutrition and other diseases axis. The percentage of correct answers was 45.6%, while the percentage of wrong answers was 54.4%. The question about celiac disease and allergy to gluten was the most often correctly answered question by the students (80.0%), whereas the question about protein restriction in patients with acute renal failure was the most often wrongly answered question (93.0%).

Table 3 illustrates the differences between the average responses of the participants’ knowledge answers according to sex using the independent-sample test. No significant differences were found in most of the knowledge axes between male and female participants. However, there were significant differences (*p* < 0.05) in the average values of the answers between the male and the female groups for the axis of nutrition and obesity only. The mean value for males was 1.59, while the mean value for females was 1.50, showing that the male group had more correct answers than the female group.

Results from Table 4 show statistically significant differences at a level of 0.01 between the averages of male and female responses to nutritional positive and negative attitude statements. The general mean value for all of the positive expressions was 3.48 out of 5.0, which means that the average falls in the fourth category of the Likert scale. Thus, the respondents in general agreed with the positive expressions. The arithmetic means of the degrees of approval for all of the positive statements ranged between 3.01 and 4.12, which means that the average degrees of approval were between “agree” and “uncertain.”

The general arithmetic mean value for the axis of negative attitudes was 3.43 out of 5.0, which means that the average was located in the fourth category of the Likert scale. Thus, the respondents mostly selected “agree.” The arithmetic mean value of the degrees of approval ranged between 2.76 and 3.91, which means that the averages were between “agree” and “uncertain.”

There were significant differences (*p* = 0.01) between the averages of male and female responses to the negative-attitude nutritional statements. The female group had a higher arithmetic mean (3.63) than the male group (3.20). In contrast, there were no significant differences in the positive-attitude statements between males and females.

## 4. Discussion

Nutrition has become an important topic in medical education, and there are many attempts to develop medical curricula for medical students. However, despite these efforts, physicians graduating from American universities are still not confident and not convinced of the adequacy of these curricula, and they feel a lack of nutritional knowledge and are not confident to provide nutritional counseling to patients [5]. Physicians with good nutritional knowledge and who have a good personal nutritional attitude are more likely, more comfortable, and more confident to provide nutritional counseling to patients. This has been recognized by some medical schools in Australia and the US as they have added courses in nutrition and lifestyle to their core curricula to increase the nutritional health of students and future patients [12].

In our study, the results showed a clear gap between the nutritional knowledge and attitude of the intern physicians. This gap could be reduced through continuous nutritional training, workshops, and educational lectures that include encouraging medical students for proper nutritional practices. The current study differed from previous studies in these results, as in an American study conducted in 2008, it was noted that physicians’ knowledge was poor in the areas of nutrition and obesity and cardiovascular nutrition [5]. In a previous study conducted in Saudi Arabia in 2004, 75% of primary care physicians considered their nutritional information as poor [13].

The majority of the study participants (62%) had normal weight, and only 10% of the participants were obese. The results were similar to a previous study conducted in Lahore in 2012, where it was found that 60% of fourth-year medical students had normal weight and only 7% were obese [12,14]. However, a study conducted in Malaysia in the same year showed the opposite, as it found a high rate of overweight and obesity among medical students, which indicated the need to encourage a healthy diet among young adults [13,15]. Another study found that the reasons for the high rate of obesity were the difficulty of the medical profession and its negative impact on the students’ diet, as they consume a lot of fast food and randomly choose their food. The study found that more than 90% of medical students were accustomed to eating fast food [14,16].

In this study, 51% of the participants were satisfied with the medical nutrition curriculum. The satisfaction rate of the participants was similar to that in a study conducted in British Columbia, according to which more than 80% of family physicians reported that they had had inadequate nutrition education at medical school, and this poor base affected their motivation for having more training on nutrition topics [15,17]. In Lebanon, researchers explained that doctors have a positive attitude toward nutritional counseling, even though they describe their nutritional education as poor [10].

In the current study, knowledge assessment was divided into four main sections: nutrition and diabetes; nutrition and obesity; nutrition and heart diseases; and nutrition and other diseases. The nutrition and diabetes axis had the highest percentage of correct answers among the topics (55.6%). In contrast, the percentage of correct answers for the axis of nutrition and heart disease was the lowest (44.1%). For example, 81% of the physicians correctly answered the question “At what LDL cholesterol level should dietary therapy be initiated for people who are at risk of heart diseases?” In contrast, only 11% of the participants correctly answered the question “Which of the following is a common complication of prednisone therapy that may require nutritional intervention?” The correct answers were generally more frequent in male than in female participants for the majority of the axes, but the differences were slight. The differences were statistically significant only for the axis of nutrition and obesity, where the correct answers in the male group were higher than those in the female group.

The results of the current study differ from those of previous studies. In an American study conducted in 2008, it was noted that physicians’ knowledge was poor in the areas of nutrition and obesity and cardiovascular nutrition [5]. In a previous study conducted in Saudi Arabia in 2004, 75% of primary care physicians considered their nutritional information as poor [13,16]. These results are in agreement with another study conducted in Canada, where the results supported the fact that physicians need additional training in nutrition [17,18]. Contrary to the results of the current study, a study conducted in Kuwait and another one in Taiwan showed that the nutritional knowledge of female physicians was much better than that of male physicians [8,19]. In a study conducted in Italy, female medical students were 48% more likely to have a “good” knowledge score than their male peers.

Males eat fewer vegetables and more fast-food meals and pasta or rice than female students, and they skip breakfast more frequently than females. Researchers have connected these observations with the high preoccupation of women with body weight and fatty food [8,20]. Another study in northwestern Iran found that more than half of the participating medical students had poor nutritional knowledge; although the study concluded that female students had greater nutritional knowledge than males, in general, all of them had poor nutritional knowledge. The results of this study showed that nutritional information was not well communicated to medical students and students of other medical sciences, especially in the field of clinical nutrition, while they had good nutritional knowledge in the field of basic nutrition. These results are in agreement with studies conducted in Taiwan and Brazil [7,9]. Another study reported a negative correlation between the years of practice and the physicians’ knowledge [20,21].

The responses of the study participants were between “agree” and “uncertain” on the Likert scale. The average response was generally “agree,” meaning that the participants generally had a positive attitude. The attitude statements were divided into positive- and negative-attitude statements after the questionnaires had been filled in for the ease of statistical analysis. The average of the positive-attitude statements in females was higher than that in males, but the differences were slight and not statistically significant. In contrast, the mean of the negative-attitude statements in female participants was significantly higher than that in male participants.

Several studies have supported these results. For example, a study conducted in Florida reported that female physicians had a significantly more positive attitude toward nutrition than male physicians [21,22]. An Iranian study also found that 90% of the participating medical students had an appropriate attitude toward nutrition, which indicates their willingness to receive nutritional information [7,9]. However, another study found that 98% of medical students in Iran had a low attitude toward nutrition [22,23].

## 5. Study Limitations

The results of this study were affected by several limitations, most notably the COVID-19 pandemic, which had many consequences. Universities were closed and the students’ access to the hospital was limited, so we were prevented from entering hospitals to meet physician interns, which led us to stop collecting data and rely on the online survey only. Moreover, the response of the physician interns to the questionnaire was poor. For these reasons, the duration of data collection was extended to about three semesters, so students from different graduation years were enrolled in the study. A previous study suggested that the low response rate may mean that physicians are less interested in nutrition [8,23].

## 6. Conclusions

Nutrition curricula in medical schools must be improved to suit the needs of students and support their skills to make them more confident in providing nutritional advice to patients and to improve the processes of examination, diagnosis, and carrying out appropriate intervention for health conditions.

Enhancing medical curricula with better and higher-quality nutritional content, increasing the hours of nutritional education, adding some optional lectures on nutrition for doctors interested in improving their nutritional knowledge, encouraging physicians to practice proper nutritional practices with patients, and training them to practice nutritional counseling with patients properly could be significant and appropriate.

## Figures and Tables

**Table 1:** Distribution of the study participants according to the demographic variables.

Variable	Options	Number	Percentage
**Age**	22–24 years	65	65.0
	25–28 years	35	35.0
**Sex**	Male	46	46.0
	Female	54	54.0
**Marital status**	Single	93	93.0
	Married	7	7.0

**Table 2:** Distribution of study participants according to the BMI.

BMI	Male		Female	
	Number	Percentage	Number	Percentage
Underweight	3	6.5	5	9.3
Normal	24	52.2	38	70.3
Overweight	14	30.4	6	11.1
Obesity	5	10.9	5	9.3
Total	**46**	**100**	**54**	**100**

**Table 3:** Differences between the average responses of the study participants by sex and nutritional knowledge using independent-sample test.

Axis	Sex	*n*	Mean	Std. Deviation	df	T. Test Value	*p*-Value
Nutrition and diabetes	Male	46	1.57	0.16	98	0.67	0.508
	Female	54	1.55	0.17			
Nutrition and obesity	Male	46	1.59	0.22	98	2.02	0.046 *
	Female	54	1.50	0.20			
Nutrition and heart disease	Male	46	1.48	0.25	98	1.52	0.132
	Female	54	1.41	0.20			
Nutrition and some different diseases	Male	46	1.45	0.20	98	0.36	0.722
	Female	54	1.46	0.20			
General average of the nutritional knowledge axes	Male	46	1.53	0.16	98	1.32	0.189
	Female	54	1.48	0.14			

* *p* Value > (0.05)

**Table 4:** Differences between the average responses of the participants about positive and negative attitude axes based on sex using independent-sample test.

Axis	Sex	*n*	Mean	Std. Deviation	df	T. Test Value	*p*-Value
Positive attitude	Male	46	3.35	0.91	98	1.42	0.158
	Female	54	3.59	0.76			
Negative attitude	Male	46	3.20	0.85	98	2.68	0.009 **
	Female	54	3.63	0.74			

** *p*-Value (0.01).

## Data Availability

Not applicable.

## Abbreviations

LDL	Low-density lipoprotein
BMI	Body mass index
NCEP	National Cholesterol Education Program
OSA	Obstructive sleep apnea
DASH	Dietary Approaches to Stop Hypertension
JNC	Joint National Committee
GI	Gastrointestinal
GFR	Glomerular filtration rate
ARF	Acute renal failure
HD	Hemodialysis
HIV	Human immunodeficiency virus
KAU	King Abdul-Aziz University
NIPS	Nutrition in Patient Care Survey
