# Correction: Bawazir et al. Nutritional Knowledge and Attitudes among Physician Interns Graduated from King Abdul-Aziz University, Jeddah, Saudi Arabia. *Healthcare* 2022, *10*, 1788

**DOI:** 10.3390/healthcare11020155

**Published:** 2023-01-04

**Authors:** Zainab Bawazir, Amani Alrasheedi, Buthaina Aljehany

**Affiliations:** Food and Nutrition Department, Faculty of Human Sciences and Design, King Abdul Aziz University, Jeddah 23213, Saudi Arabia

In the original publication [1], the funder **Deanship of Scientific Research of King Abdul-Aziz University, D-1025-253-1443**, was not included.

A correction has also been made to the ***Results*** section, ***Paragraph Number 8***, where the word “positive” has been corrected to “negative”:

**The general arithmetic mean value for the axis of negative attitudes was 3.43 out of 5.0, which means that the average was located in the fourth category of the Likert scale. Thus, the respondents mostly selected “agree.” The arithmetic mean value of the degrees of approval ranged between 2.76 and 3.91, which means that the averages were between “agree” and “uncertain”.**

Another correction has also been made to ***Paragraph Number 2*** from the ***Results*** section, where duplicated paragraph was removed:

**Knowledge assessment was divided into four main sections: nutrition and diabetes; nutrition and obesity; nutrition and heart diseases; and nutrition and other diseases. The study participants’ responses to the questions on the nutrition and diabetes axis showed that correct answers accounted for 55.6% of the questions, and wrong answers accounted for 44.4%, which illustrated the difference in their knowledge about this subject. The question that most of the participants answered correctly (81.0%) was about the complication of prednisone therapy that requires nutritional intervention. Most of the students (76.0%) did not know the correct answer to the question on how many grams of carbohydrate are contained in one serving unit.**

The authors state that the scientific conclusions are unaffected. This correction was approved by the Academic Editor. The original publication has also been updated.
